# Jiangtang Decoction for Type 2 Diabetes and NAFLD: Integrative Analysis *via* Network Pharmacology, Mendelian Randomization, Molecular Docking, and *In Vitro* Validation

**DOI:** 10.2174/0118715303424915250818045151

**Published:** 2025-08-22

**Authors:** Wenbo Gong, Xueke Lu, Siying Weng

**Affiliations:** 1 Ningbo Municipal Hospital of TCM, Affiliated Hospital of Zhejiang Chinese Medical University, Ningbo, China;; 2 The Affiliated Joint Training Base of Zhejiang Chinese Medical University, Ningbo, China

**Keywords:** Jiangtang Decoction, type 2 diabetes mellitus, non-alcoholic fatty liver disease, network pharmacology, causal inference, mendelian randomization, molecular docking

## Abstract

**Introduction:**

Jiangtang Decoction (JTD) demonstrates notable efficacy in managing Type 2 Diabetes Mellitus (T2DM) and Non-Alcoholic Fatty Liver Disease (NAFLD). This study aimed to elucidate JTD's causal targets and therapeutic mechanisms by integrating network pharmacology, Summary-data Mendelian Randomization (SMR), and molecular docking, complemented by *in vitro* validation.

**Materials and Methods:**

JTD's targets were cross-matched with genes associated with T2DM or NAFLD. SMR and co-localization analyses, utilizing eQTL and pQTL data, identified causal signals for each disease and their shared counterparts. GO/KEGG enrichment analyses were performed on these shared signals. Molecular docking assessed binding interactions. *In vitro* experiments, including ELISA (Enzyme-linked immunosorbent assay) and lipid staining, evaluated JTD's hypoglycemic/hypolipidemic effects, while PCR/immunofluorescence validated key molecular predictions in High-Glucose and High-Lipid (HGHL)-induced HepG2 cells.

**Results:**

Initial network pharmacology identified 1107 JTD targets for T2DM and 1126 for NAFLD. SMR analyses revealed 78 eQTLs and 67 pQTLs for T2DM, and 40 eQTLs and 38 pQTLs for NAFLD. Eight shared causal genetic signals were identified: *ADAMTS4, ALDH2, GLO1, CDH1, PDHB, PRKAB1, TCF4*, and *EP300*. *In vitro*, JTD significantly attenuated hepatic gluconeogenesis and lipid deposition, downregulated IL-1β, and notably restored PRKAB1 expression in HepG2 cells treated with HGHL.

**Discussion:**

Enrichment analysis revealed shared processes, including membrane microdomains, oxidoreductase activity, and responses to nutrient and oxygen levels. PRKAB1 exhibited a strong binding affinity with the JTD component Salsalate.

**Conclusion:**

This study identified eight genes that are genetically predicted to be causal for the therapeutic effects of JTD on both T2DM and NAFLD, thereby establishing a genetic link between the conditions. These targets and associated pathways illuminate common underlying mechanisms, supporting JTD's potential for integrated treatment strategies and providing a basis for further investigation.

## 1 INTRODUCTION

Type 2 Diabetes Mellitus (T2DM), constituting 90–95% of all diabetes cases, is projected to affect 693 million adults globally by 2045 [1, 2]. This condition is defined by Insulin Resistance (IR) and dysfunctional insulin release, resulting in elevated blood glucose levels and associated complications, including Non-Alcoholic Fatty Liver Disease (NAFLD) [1, 3]. NAFLD is a condition that affects roughly one-quarter of the world's population. It is a persistent inflammatory condition affecting the liver, characterized by the presence of hepatic fat deposition, and may progress to cirrhosis and hepatocellular carcinoma [4]. Both T2DM and NAFLD share common pathogenic mechanisms, including insulin resistance, chronic inflammation, and dysfunctions in lipid metabolism [5, 6]. Despite their significant global health burden, effective treatments for these conditions remain limited. Current T2DM management focuses on glycemic control through lifestyle changes, medications, and insulin therapy; however, these approaches often fail to address the underlying pathophysiology or prevent complications [7, 8]. For NAFLD, no specific pharmacological treatments are available, and management relies on lifestyle interventions [4]. Therefore, there is an imperative need for enhanced and tailored therapeutic approaches.

Traditional Chinese Medicine (TCM) is essential in the treatment of complex ailments [9-11]. Jiangtang Decoction (JTD), a classical formula primarily composed of six medicinal herbs (*Atractylodes macrocephala, Scrophularia ningpoensis, Astragalus membranaceus, Rehmannia glutinosa, Pueraria lobata*, and *Salvia miltiorrhiza*), is commonly used for its antihyperglycemic effects. Recent studies, supported by clinical evidence, have highlighted the significant role of JTD in diabetes management. It has demonstrated potential in reducing glucose levels, modulating lipid metabolism, improving insulin sensitivity, and mitigating oxidative stress, suggesting its value as a complementary approach for prediabetes [12]. Notably, *Atractylodes macrocephala*, a key component of JTD, is known to regulate diabetes by interacting with signaling pathways such as AGE-RAGE, PI3K-Akt, and RPTK, thereby reducing apoptosis, inflammation, and insulin resistance [13]. Given its regulatory role in metabolic dysfunction, JTD demonstrates potential therapeutic effects on NAFLD. For instance, Jiangtang Qingre Formula, an adaptation of JTD, has been shown to mitigate NAFLD by regulating immune cell populations and inducing anti-inflammatory and anti-fibrotic actions [14]. Despite its promising therapeutic potential for both T2DM and NAFLD, the precise underlying mechanisms of JTD remain to be fully elucidated.

Given the shared pathogenesis of T2DM and NAFLD, investigating the common therapeutic mechanisms of JTD is crucial. As a compound herbal formulation, JTD contains multiple active components targeting various pathways. However, their precise interactions and contributions to ameliorating T2DM or NAFLD are not fully understood. Therefore, a comprehensive analysis of JTD's target genes is essential for elucidating its molecular mechanisms and establishing a scientific basis for its therapeutic application. Network pharmacology, employing bioinformatics and systems biology, can explore drug-molecule interactions to identify potential targets and mechanisms [14, 15]. However, establishing robust causal links solely through network pharmacology can be challenging. Summary-data-based Mendelian Randomization (SMR) provides a method for inferring causality by integrating information across biological domains, such as genomics and proteomics, leveraging large GWAS datasets to offer robust genetic insights [16-18]. SMR analysis infers potential causal relationships by integrating genomic data, and its conclusions depend on key assumptions, such as the absence of horizontal pleiotropy, which requires further validation through biological experiments. Consequently, combining network pharmacology with SMR provides a powerful, evidence-based approach for precision target identification [19, 20]. Building on this, the current research employed network pharmacology to identify JTD's potential targets and SMR analysis to investigate their causal relationships with T2DM and NAFLD risk. Common candidate causal genes were subsequently identified and subjected to enrichment analysis. Molecular docking was used to forecast the interactions between the components of JTD and the key target proteins. Finally, *in vitro* experiments were conducted to validate key findings. This research aims to identify new therapeutic targets and facilitate the development of precise treatment strategies for T2DM and NAFLD.

This study employed network pharmacology to identify JTD target genes and SMR analysis to investigate their causal relationships with T2DM and NAFLD risk. Common genes were identified and analyzed for enrichment. Molecular docking confirmed interactions between JTD components and key target proteins. The research aims to uncover new therapeutic targets and support precise treatment strategies for T2DM and NAFLD.

## 2 MATERIALS & METHODS

### 2.1 Study Design

Potential target genes of JTD were identified using publicly available databases. The ADME properties of these targets were evaluated using SwissADME (http://www. swissadme.ch/). T2DM- and NAFLD-associated genes from CTD (https://www.omim.org/), MalaCards (https://www.malacards.org/), and GeneCards (https://www.genecards.org/) were compared with JTD target genes to identify overlaps. SMR and colocalization analyses were used to assess the relationships between these genes and disease risks. Their functions were elucidated through GO and KEGG analyses. A drug-target network was constructed based on genes associated with both conditions. Molecular docking confirmed interactions between JTD elements and primary target proteins. *In vitro* experiments confirmed that JTD exhibits hypoglycemic, hypolipidemic, and anti-inflammatory effects. Additionally, the results of the molecular docking analysis were validated to support these findings.

### 2.2 Identification of JTD Components and Target Gene Prediction

Active components of JTD were retrieved from the TCMSP database (https://old.tcmsp-e.com/tcmsp.php) and the BATMAN-TCM platform (http://bionet.ncpsb.org. cn/batman-tcm/#/home). The ADME properties of these components were assessed using SwissADME, retaining only those with gastrointestinal absorption and a bioavailability score greater than 0.5, and meeting at least two drug-likeness criteria [21]. Targets were identified from TCMSP and BATMAN-TCM, with additional predictions from SEA (https://sea.bkslab.org/) for compounds without known targets (Max Tc > 0.5, *p*-value < 0.05) [22]. After removing duplicates and applying ADME filters, the final set of active ingredients and targets was identified for further analysis.

### 2.3 Acquisition of T2DM or NAFLD-related Genes

Genes implicated in T2DM or NAFLD were retrieved from CTD, MalaCards, and GeneCards databases. In CTD, genes with direct evidence and a top 25% Inference Score were selected. In GeneCards, only the top 10% of protein-coding genes related to T2DM were retained. Following the merger and elimination of duplicates, a comprehensive set of T2DM- and NAFLD-associated genes was obtained for analysis.

### 2.4 Data Sources

The T2DM dataset from FinnGen comprised 82,878 cases and 403,489 controls, while the NAFLD dataset contained 3,504 cases and 496,844 controls, all of whom were of European ancestry. Blood gene eQTL summary statistics were obtained from the eQTLGen consortium (31,684 individuals) [23]. Genetic links with circulating protein levels were identified in three separate pQTL studies, comprising 10,708 Europeans from the Fenland database [24], 54,219 Europeans from UKB-PPP [25], and 35,559 Icelanders from DECODE [26].

Detailed information for each outcome is provided in Supplementary Table **1**. This study utilized summary statistics from publicly available GWAS studies, for which ethical approval had already been obtained, thereby eliminating the need for additional ethical approval.

### 2.5 Summary-data-based MR Analysis

The SMR methodology combines GWAS data with eQTL and pQTL statistics to investigate gene expression, protein levels, and the risk of T2DM and NAFLD [27]. By leveraging top-associated *cis*-QTLs, SMR significantly boosts statistical power. *Cis*-QTLs were identified within a ±1000 kb region adjacent to the gene of interest (*P*< 5.0×10^−8^) [28]. SNPs with allele frequency differences exceeding 0.2 across datasets were excluded. To differentiate between pleiotropy and linkage, the Heterogeneity in Dependent Instruments (HEIDI) test was employed. Variants with potential pleiotropy (a P-HEIDI value < 0.05) were excluded from further analysis. Associations meeting the criteria (P SMR < 0.05 and P-HEIDI > 0.05) were selected for co-localization analysis in QTL datasets.

### 2.6 Co-localization Analysis

Co-localization analysis was performed to determine whether eQTLs, pQTLs, and T2DM/NAFLD risk factors share a common causal variant. The study utilized a Bayesian method to integrate information from these signals, thereby enhancing the precision of identifying the causal variant [29]. These analyses report five different posterior probabilities: H0-H4 [29]. When GWAS signals coincide with QTLs, it implies that the GWAS locus might impact the complex trait or disease phenotype by modulating gene expression [30, 31]. For colocalization analysis, all SNPs located within a 1000 kb distance upstream and downstream of each top eQTL and pQTL were extracted to calculate the posterior probability of H4 (PPH4). Strong evidence of colocalization was considered when the PPH4 value exceeded 0.5 with the P12 parameter set to 5 × 10^5, and simultaneously, the PPH3 value was less than 0.5 with the P12 parameter set to 1 × 10^5 [32].

### 2.7 Enrichment Analysis of PPI Networks

The Biological Processes (BP), Molecular Functions (MF), and Cellular Components (CC) of the genes associated with T2DM or NAFLD were explored through Gene Ontology (GO) enrichment analysis. Additionally, key pathways associated with these hub genes were identified *via* KEGG analysis, with significance determined at an adjusted *P* < 0.05 using the Benjamini-Hochberg method approach.

### 2.8 Molecular Docking

The 3D structure files of JTD components were obtained from the PubChem database and converted using Open Babel 2.4.1 for use as ligands. Meanwhile, PDB files of the target proteins were retrieved from the PDB and preprocessed in PyMOL 2.6.0 by removing water molecules and ions and adding hydrogen atoms. Both the JTD compounds and the protein structures were then subjected to molecular docking analysis using AutoDock Vina. The docking results were visualized using PyMOL. Based on established criteria, a ligand-target binding affinity of less than -4.25 kcal/mol indicates binding activity. Values lower than -5.0 kcal/mol imply favorable binding activity, while results below -7.0 kcal/mol denote robust binding activity [33].

### 2.9 Preparation of JTD

*Astragalus membranaceus* (Gansu, China; batch 226942), *Atractylodes lancea* (Liaoning, China; batch 214463), *Rehmannia glutinosa* (Henan, China; batch 252375), *Pueraria lobata* (Henan, China; batch 221765), *Scrophularia ningpoensis* (Zhejiang, China; batch 217684), and *Salvia miltiorrhiza* (Zhejiang, China; batch 215139) were sourced from Zhejiang Chinese Medical University Herbal Pieces Co., Ltd. All herbs passed quality testing per the 2015 Chinese Pharmacopoeia and TS-ZL-03 standards. JTD formula includes: *Astragalus membranaceus* (30 grams), *Atractylodes lancea* (20 grams), *Rehmannia glutinosa* (20 grams), *Pueraria lobata* (20 grams), *Salvia miltiorrhiza* (20 grams), and *Scrophularia ningpoensis* (10 grams). The herbs were ground, mixed with 500 grams of water, and boiled for 1 hour [32]. The pH of the filtrate was adjusted to 7.0 using sodium citrate and sodium carbonate. The concentration of the crude extract was 3 g/mL. The JTD liquid was freeze-dried using a pharmaceutical vacuum freeze-dryer through the following process: pre-freezing at -50°C for 4 hours, sublimation drying at -20°C for 12 hours, and final drying at 32°C for 8 hours. The resulting powder was stored at 4°C. Before use, the sample was dissolved in medium, centrifuged at 4000×g for 30 minutes, and the supernatant was collected for further experiments.

### 2.10 Establishment of High-Glucose and High-Lipid (HGHL) Cell Model

HepG2 cells (No. SCSP-510) were obtained from the National Collection of Authenticated Cell Cultures (Shanghai, China). Derived from a 15-year-old Caucasian male with hepatocellular carcinoma in 1975, these cells exhibit key features including hepatocyte-specific secretory functions, stable *in vitro* proliferation, and malignant phenotypes, making them a standard model in liver cancer research. The cells were cultured in DMEM medium supplemented with 10% fetal bovine serum (Gibco, California, USA) under standard conditions (37°C, 5% CO_2_, saturated humidity). The cell line was passaged to the third generation, and experiments were performed at 90% confluence. In subsequent experiments, the glucose concentration was adjusted to 16.7 mmol/L, and a mixture of sodium palmitate (500 μM)/sodium oleate (250 μM) (SYSJKJ001, Kunchuang Bio, Xi'an, China) was added. The cells were cultured for 48 hours to establish the HGHL model, confirmed by experimental criteria. The model cells were treated with JTD powder (low and high concentrations). A positive drug control group received Metformin (150 μmol/L; M107827, Aladdin, Shanghai, China). Untreated cells served as the model group, and untreated HepG2 cells under normal conditions served as the control group.

### 2.11 Cell Counting Kit-8 (CCK-8)

The effect of YD concentration on cell proliferation was assessed using a CCK8 assay (CK04, Dojindo Laboratories, Japan). HepG2 cells were plated in 96-well plates and incubated for 48 h. JTD freeze-dried powder was added at concentrations of 0.01%, 0.1%, 0.5%, 1%, 10%, and 100%. After 48 hours, 10 μL of CCK-8 solution was added to each well. Plates were incubated in the dark for 1 hour, and the absorbance was measured at 450 nm using a microplate reader. Cell viability (%) was calculated as: (OD value of experimental group / OD value of control group)×100%.

### 2.12 Nile Red Staining for Lipid Fluorescence

The lipid fluorescence staining kit (G1264, Solarbio, China) was applied for analysis. HepG2 cells were cultured in 6-well plates at 37°C with 5% CO_2_ for 2 days. Cells were washed with PBS, fixed with 1 mL of fixation solution for 15 minutes, and then aspirated. Following PBS washing, the staining solution was added and incubated in the dark at room temperature for 10 min. After gentle shaking, the cells were rinsed with 1 mL of 1× PBS, retaining the final rinse solution. Cells were stored in the dark and observed under a fluorescence microscope (BX53, Olympus, Japan) within 30 min (excitation: 543 nm, emission: 598 nm).

### 2.13 Glucose Production

HepG2 cells were seeded into 6-well plates. Sodium pyruvate (2 mmol/L) and sodium lactate (20 mmol/L) were added to the cells in all groups. Glucagon (100 nmol/L) was specifically supplemented to the cells in 4 experimental groups. After 6 hours of incubation, the glucose content in the supernatant was determined using a glucose oxidase assay kit (BC0690, Solarbio, Beijing, China). To accurately normalize glucose levels, the protein concentration in each sample well was evaluated using the BCA method, ensuring precise quantification of glucose production relative to cellular protein content.

### 2.14 ELISA

Following the instructions for the cyclic Adenosine Monophosphate (cAMP) kit (EH4274, FineTest, Wuhan, China) and Interleukin-1β (IL-1β) kit (FNab10305, FineTest, Wuhan, China), cAMP and IL-1β levels in the supernatant of HepG2 cell were quantified by ELISA. After intervention, cells were gathered, spun at 1000×g for 5 minutes, and then reconstituted in PBS with 1% PMSF added. The cells were broken down using freeze-thaw cycles and subsequently spun at 1500×g for 10 min within a temperature range of 2-8°C. The absorbance of the supernatants was determined at 450 nm with a microplate reader for analysis. All the steps that were followed were in accordance with the manufacturer's instructions.

### 2.15 qPCR

After the intervention, total RNA was extracted from HepG2 cells using Trizol reagent. Reverse transcription was performed with the ReverTra Ace qPCR RT Kit (East Asian Textile Life Sciences, Japan). PCR amplification (20 μl) consisted of pre-denaturation at 94°C for 3 min, followed by 40 cycles of denaturation at 94°C for 10 s and annealing/extension at 60°C for 30 s. Relative gene expression was calculated using the 2^-ΔΔ^Ct method, normalized to Actin. PCR analyzed PRKAB1 mRNA levels on the ABI PRISM®7900 HT system. Primers were designed and synthesized by Wuhan GENE CREATE Bio Co., Ltd., with sequences in Table **1**.

### 2.16 Immunofluorescence

Cells were cultured on coverslips. Following treatment, the culture medium was discarded, and the cells were fixed with 4% paraformaldehyde for 15 minutes, permeabilized with 0.2% Triton X-100 for 20 minutes, and then blocked with 3% BSA at 37°C for 30 minutes. Primary antibody (rabbit anti-PRKAB1, 1:300) was added and incubated overnight at 4°C. The slides were then incubated with the secondary antibody (red-fluorescent goat anti-rabbit IgG, 1:200) for one hour. Finally, nuclei were labeled with DAPI by incubating the samples for five minutes in the dark. Samples were mounted with an anti-fade medium and imaged under a fluorescence microscope (BX53, Olympus, Japan).

### 2.17 Statistical Analysis

Statistical analyses were performed using R (v4.3.0). Forest plots were generated with the “forestplot” package, and GO/KEGG enrichment analyses were conducted with the “Clusterprofiler” package. Data are presented as mean ± SD. Group differences were evaluated by one-way ANOVA followed by Tukey’s post hoc test for pairwise comparisons. A *p*-value < 0.05 indicated statistical significance. SPSS 23.0 was used for statistical analyses, and figures were created with Prism 6.02 and Illustrator CS6.

## 3 RESULTS

### 3.1 Potential Targets of JTD in T2DM or NAFLD Therapy

Utilizing SwissADME and established drug-likeness criteria, 430 active components were identified from JTD (Supplementary Table **2**), corresponding to 1,416 predicted target genes (Supplementary Table **3**). Additionally, the study identified 9,153 genes associated with T2DM and 9,640 genes associated with NAFLD using data from the CTD, MalaCards, and GeneCards databases (Supplementary Table **4**). By intersecting JTD's predicted targets with these disease-related genes, researchers found 1,107 common genes for T2DM and 1,126 for NAFLD. A further intersection analysis of these common genes revealed 1,005 genes potentially shared among all JTD targets, T2DM-related genes, and NAFLD-related genes. This group of 1,005 shared genes then formed the gene pool for the subsequent causal inference analyses (Supplementary Fig. **1**; Supplementary Table **5**).

### 3.2 Causal Links between Target Gene Expression, Protein Levels, and T2DM Risk

**Fig. (1):**
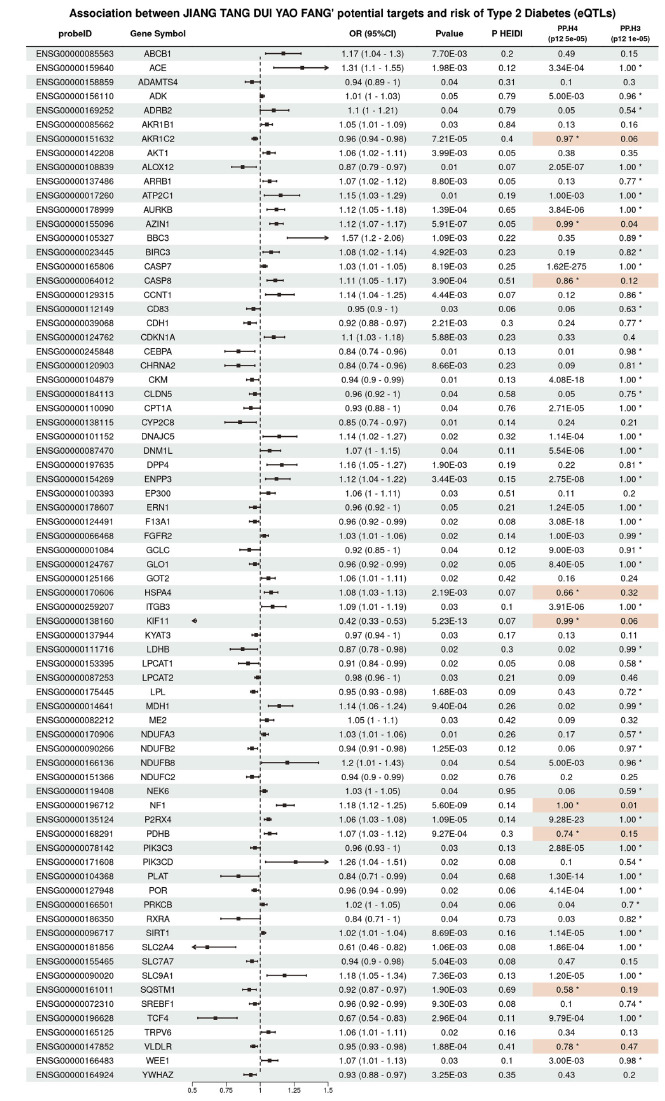
SMR analyses of the causal effects of common gene expressions on T2DM. Forest plot depicting the association between representative gene expressions and T2DM. OR: Odds ratio. **Abbreviations:** CI: Confidence interval. Genes associated with T2DM were highlighted in orange.

From the 1,005 shared genes, 709 were testable in the eQTLGen database. SMR analysis identified 73 target genes whose expression levels were associated with T2DM risk (P SMR < 0.05 and P-HEIDI > 0.05) [34], of which 42 exhibited positive associations (OR > 1) (Fig. **1**; Supplementary Table **6**). Strong colocalization evidence (PPH4 > 0.5 & PPH3 < 0.5) for 9 of these eQTL signals, such as for *PDHB* (Supplementary Fig. **2A**).

**Fig. (2):**
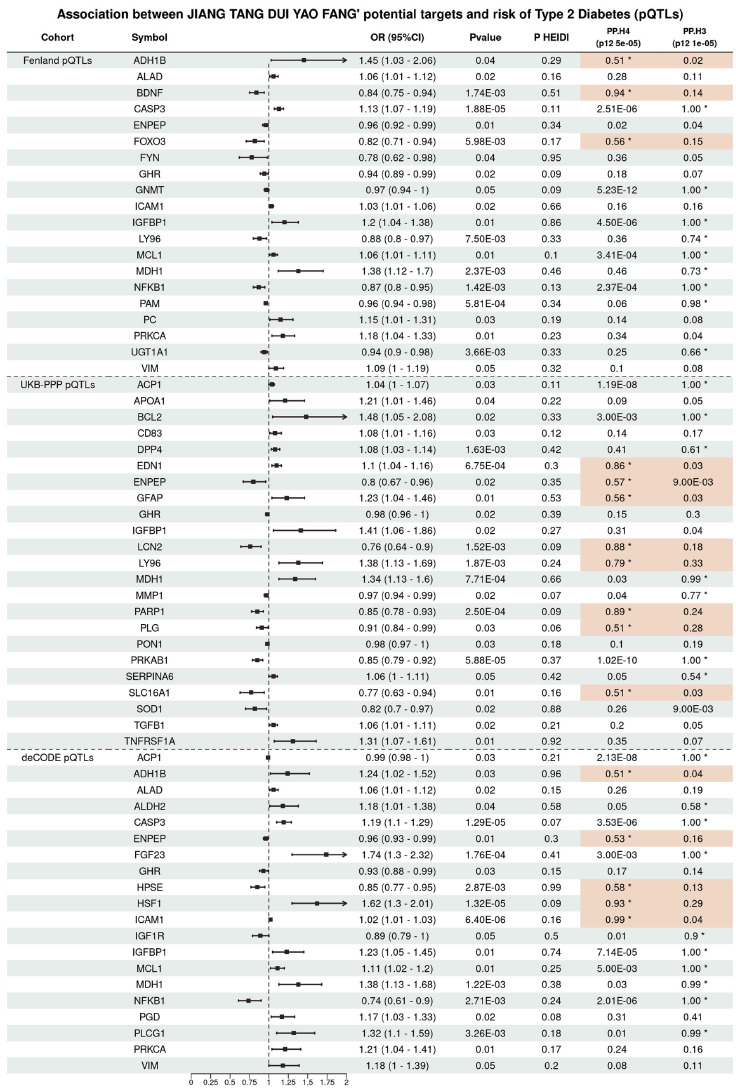
SMR analyses of the causal effects of common protein levels on T2DM. Forest plot depicting the association between representative protein abundance and T2DM. OR: Odds ratio. **Abbreviations:** CI: Confidence interval. Proteins associated with T2DM were highlighted in orange.

At the protein level, SMR analysis across the Fenland, UKB-PPP, and DECODE pQTL datasets identified 63 unique proteins causally associated with T2DM risk (P SMR < 0.05 and P-HEIDI > 0.05) (Fig. **2**; Supplementary Table **7**). Among these, 14 proteins showed strong colocalization evidence, for example, *ADH1B* (Supplementary Fig. **2B**). Cumulatively, 115 distinct genetic signals (eQTLs and pQTLs) were found to be causally linked to T2DM risk.

### 3.3 Causal Links between Target Gene Expression and Protein Levels on NAFLD Risk

**Fig. (3):**
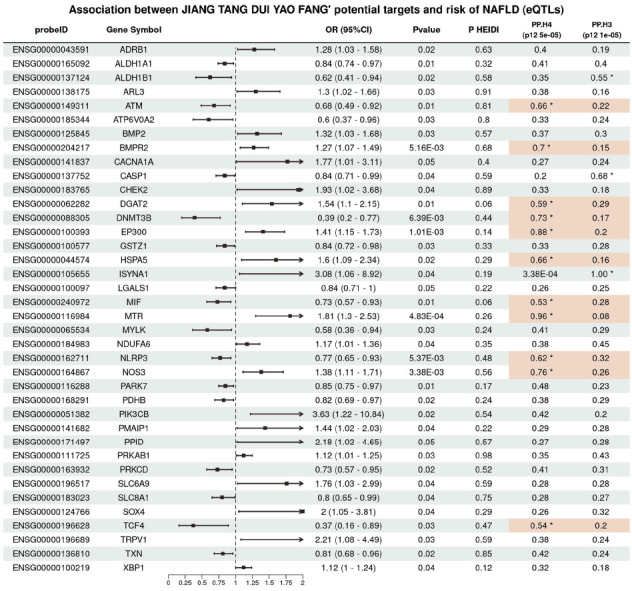
SMR analyses of the causal effects of common gene expression on NAFLD. Forest plot depicting the association between representative gene expressions and NAFLD. **Abbreviations:** OR: Odds ratio. CI: Confidence interval. Proteins associated with T2DM were highlighted in orange.

For NAFLD, SMR analysis of the 709 testable genes identified 38 target genes whose expression levels were associated with NAFLD risk (P SMR < 0.05 and P-HEIDI > 0.05) (Fig. **3**; Supplementary Table **8**). Strong colocalization evidence supported 11 of these signals, including *EP300* (Supplementary Fig. **3A**).

**Fig. (4):**
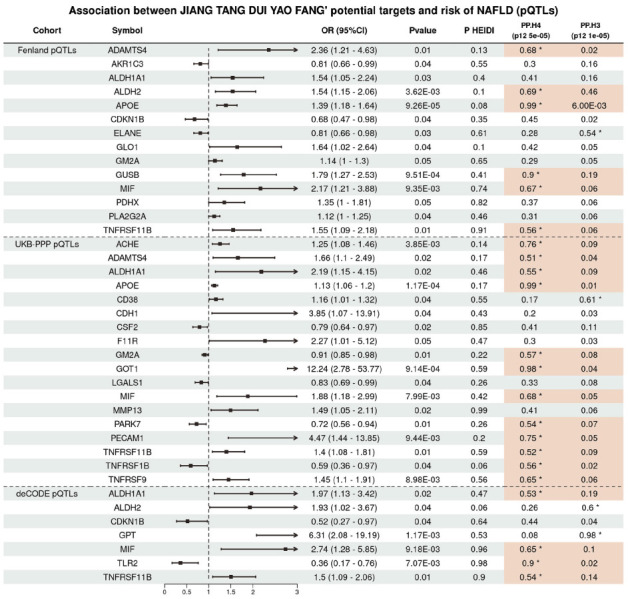
SMR analyses of the causal effects of common protein levels on NAFLD. Forest plot depicting the association between representative protein abundance and NAFLD. **Abbreviations:** OR: Odds ratio. CI: Confidence interval. Proteins associated with T2DM were highlighted in orange.

At the protein level, SMR analysis across the three pQTL datasets identified a total of 33 unique proteins associated with NAFLD risk (P SMR < 0.05 and P-HEIDI > 0.05) (Fig. **4**; Supplementary Table **9**). Strong colocalization evidence was found for 15 proteins, including *ADAMTS4* (Supplementary Fig. **3B**). In total, 62 distinct genetic signals showed causal links to NAFLD risk.

### 3.4 Integration of Signals Causally Associated with T2DM or NAFLD

**Fig. (5):**
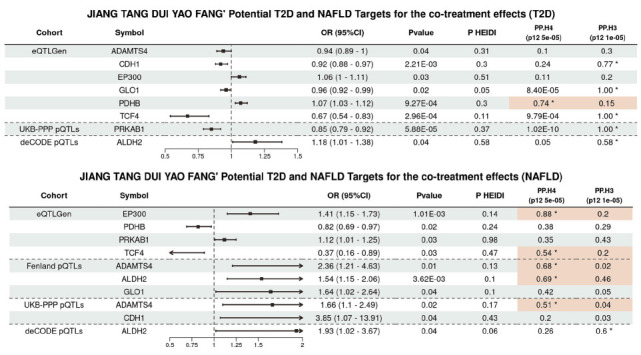
SMR analyses of the causal effects of 8 common signals on T2DM or NAFLD. Forest plot depicting the association between the 8 common signals and T2DM (top panel) and NAFLD (lower panel). **Abbreviations:** lipid fluorescence compared to OR: Odds ratio. CI: Confidence interval. Proteins associated with T2DM were highlighted in orange.

Integration of the SMR results for T2DM and NAFLD identified eight genes (*ADAMTS4, ALDH2, GLO1, CDH1, PDHB, PRKAB1, TCF4*, and *EP300*) (Fig. **5**). This study identified eight shared genes through a rigorous process of independent analysis and intersection validation. For T2DM, SMR and co-localization analyses selected 115 candidate causal genes from 1,005 candidates (78 eQTLs + 67 pQTLs, all meeting P SMR < 0.05, P-HEIDI > 0.05, and PPH4 > 0.5). Using the same criteria, 62 candidate causal genes (40 eQTLs and 38 pQTLs) were identified for NAFLD. The eight shared genes were identified by comparing the two gene sets. This method ensures that each gene was independently validated in both diseases, rather than through combined analysis. These genes represent high-confidence, shared molecular targets that potentially mediate JTD's therapeutic effects.

### 3.5 Enrichment Analysis

**Fig. (6):**
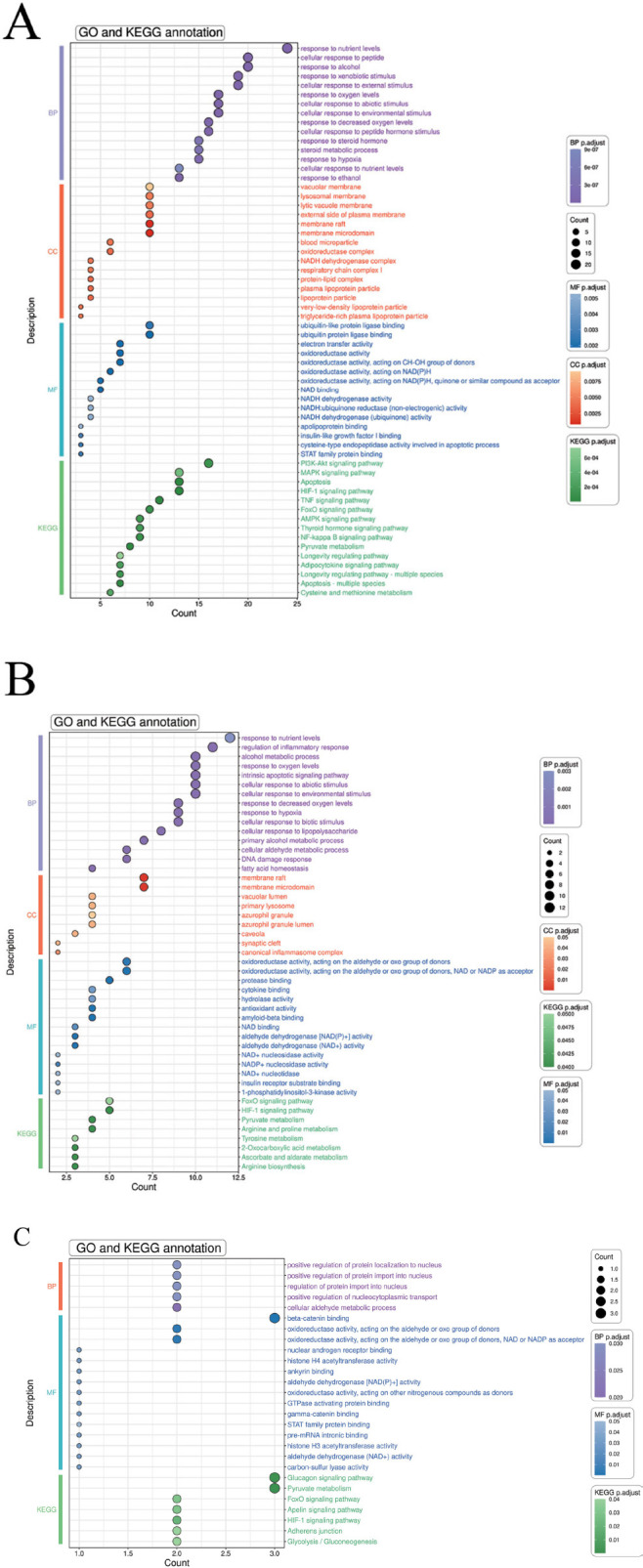
Enrichment analysis of JTD target genes associated with T2DM or NAFLD. GO and KEGG analysis of JTD target genes associated with (**A**) T2DM or (**B**) NAFLD. (**C**) The enrichment analysis of the 8 common target genes associated with both conditions. The size of the bubbles represented the gene counts involved in each term, while the colors represented the adjusted p-value.

In order to examine the biological significance of the causally implicated genes for T2DM or NAFLD, GO and KEGG pathway analyses were conducted. For T2DM-associated genetically predicted causal genes (Fig. **6A**), significant enrichment was observed in KEGG pathways, such as PI3K-Akt, MAPK, apoptosis, HIF-1, AMPK, and NF-κB signaling, as well as in GO Biological Processes (BP), including responses to nutrient levels and cellular reactions to peptides. Similarly, NAFLD-associated genetically predicted causal genes (Fig. **6B**) were implicated in BPs such as response to nutrient levels and regulation of inflammatory response, and pathways including FoxO and HIF-1 signaling. Notably, GO terms such as 'membrane microdomain', 'oxidoreductase activity', 'response to nutrient levels', and 'response to decreased oxygen levels' were found to be common among the enriched terms for T2DM and NAFLD genetically predicted causal genes, suggesting shared pathogenic mechanisms. Furthermore, enrichment analysis of the eight common genetically predicted causal genes (Fig. **6C**) revealed significant involvement of the glucagon signaling pathway, pyruvate metabolism, and BPs like protein import into the nucleus, indicating their potential contribution to the shared pathophysiology.

### 3.6 Network Pharmacology Analysis

**Fig. (7):**
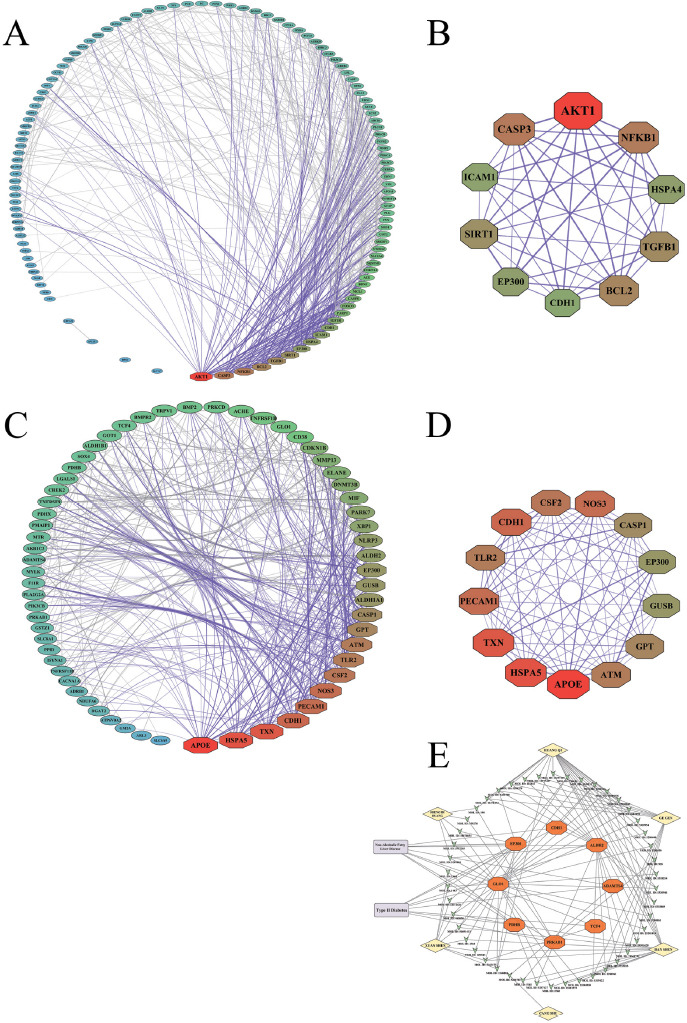
Network pharmacology analysis. (**A**) The PPI network of the 115 causal targets associated with T2DM; (**B**) The interaction of the 10 hub targets associated with T2DM; (**C**) The PPI network of the 62 causal targets associated with NAFLD; (**D**) The interaction of the 13 hub targets associated with NAFLD. Key hub nodes were marked with octagons, while ellipses indicated other candidate target nodes. (**E**) The network of disease-herb-compounds-target genes. The size and color shades of the nodes reflected their degree. Interactions were indicated as purple lines. Interactions were indicated as purple lines.

Through integration, a total of 115 eQTL or pQTL signals associated with T2DM were utilized to construct a PPI network (Figs. **7A**-**B**). The top 10 hub targets identified were *AKT1, BCL2, CASP3, CDH1, EP300, HSPA4, ICAM1, NFKB1, SIRT1* and *TGFB1*. Similarly, the PPI network of the 62 signals associated with NAFLD was constructed, identifying 13 hub targets, including APOE, ATM, CASP1, CDH1, CSF2, EP300, GPT, GUSB, HSPA5, NOS3, PECAM1, TLR2, and *TXN* (Figs. **7C**-**D**). The T2DM/NAFLD-JTD components-genes network was constructed based on the identified genes associated with both conditions. (Fig. **7E**) illustrates the complex interactions among 6 herbs, 46 compounds, and 8 target genes. This graphical depiction emphasizes the therapeutic significance of these genes as potential targets for JTD in addressing T2DM and NAFLD.

### 3.7 Molecular Docking Validation

**Fig. (8):**
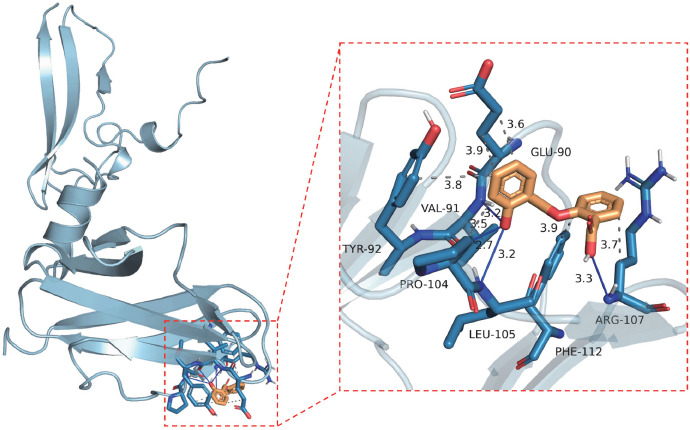
Molecular docking validation. Molecular dynamics simulations illustrate the interaction between Salsalate and PRKAB1.

Molecular docking analysis was performed on the eight shared, genetically predicted causal genes/proteins, along with the selected active ingredients of JTD, to investigate their interaction potential further. Binding affinities are detailed in Supplementary Table **10**. (Fig. **8** illustrates the predicted strong interaction between PRKAB1 and Salsalate (a key JTD ingredient), with a binding affinity of -7.043 kcal·mol ^-1^.

### 3.8 Effect of JTD on Cell Viability

**Fig. (9):**
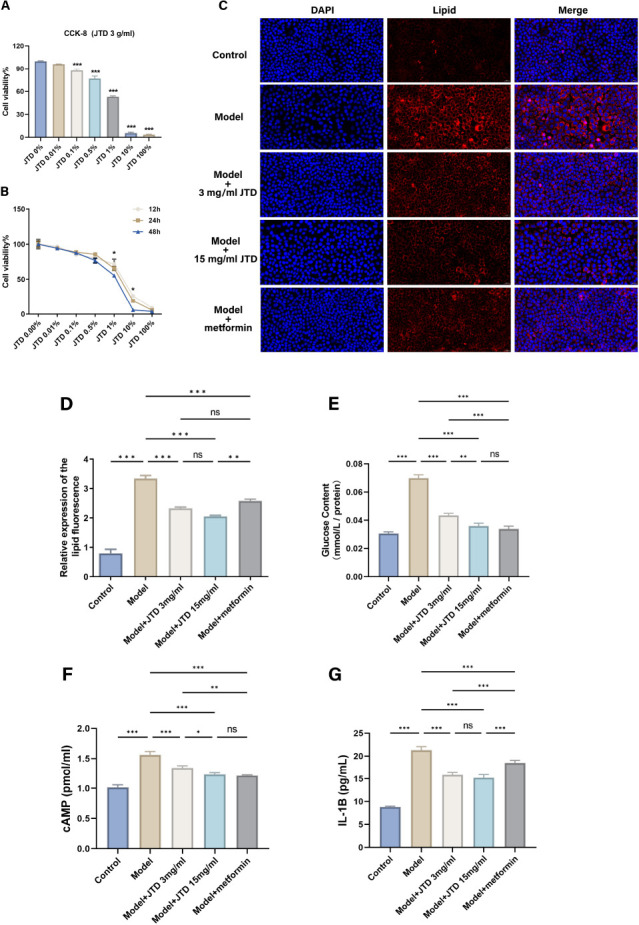
Hypoglycemic and hypolipidemic effects of JTD *in vitro*. The CCK-8 method was employed to determine the suitable concentration (**A**) and optimal timing (**B**) for JTD intervention. JTD treatment resulted in reduced lipid accumulation in hepatic cells (C-D), attenuated hepatic gluconeogenesis (**E-F**), and lowered cellular inflammatory cytokine levels (**G**). *p < 0.05, **p < 0.01, ***p < 0.001.

To determine the optimal JTD concentration for HepG2 cells, a CCK-8 assay was performed. After 48 h, the 0.01% group did not show any significant changes, while cell survival decreased significantly at concentrations above 0.1% (Fig. **9A**). Furthermore, after treating cells with JTD for 12 h, 24 h, and 48 h, the cell survival of the 0.5% JTD group was found to be higher at 24 h than at both 12 h and 48 h, although no significant differences were detected among the time points (Fig. **9B**). Based on these results, subsequent experiments used JTD concentrations of 0.1% (3 mg/mL) and 0.5% (15 mg/mL) with an intervention time of 24 h.

### 3.9 JTD Reduces Lipid Deposition in HGHL HepG2 Cells

The results revealed that the control group exhibited weak lipid fluorescence intensity and low lipid accumulation in HepG2 cells. In contrast, the model group showed significantly enhanced lipid fluorescence (*P* < 0.001), indicating substantial lipid accumulation. The JTD groups (3 mg/mL and 15 mg/mL) and the Met-treated group exhibited reduced lipid fluorescence compared to the model group (*P* < 0.001). No significant differences in lipid fluorescence intensity were observed between the two JTD treatment groups. The Metformin-treated group exhibited higher lipid fluorescence compared to the JTD group (15 mg/mL) but showed a similar level to the JTD group (3 mg/mL) (Figs. **9C**-**D**). These results indicate that JTD exhibits a lipid-lowering effect in HGHL-induced HepG2 cells, similar to metformin, suggesting its potential role in regulating liver metabolism. However, confirmation in models that more closely resemble physiological conditions is needed.

### 3.10 JTD Reduces Glucose Production in HGHL HepG2 Cells

The glucose content in HepG2 cells treated with JTD (3 mg/mL and 15 mg/mL) was measured using a glucose oxidase assay kit. The results demonstrated that the model group had significantly higher glucose levels than the normal group (*P* < 0.001). Both JTD groups (3 mg/mL and 15 mg/mL) and the Met group showed reduced glucose levels compared to the model group (*P* < 0.001). While the 15 mg/mL JTD group showed a trend towards lower glucose levels compared to the 3 mg/mL JTD group, the difference did not reach statistical significance (Fig. **9E**). These results show that JTD could significantly suppress glucose production in HepG2 cells induced by HGHL. The inhibitory effect of a high concentration of JTD was similar to that of metformin.

### 3.11 JTD Reduces cAMP and IL-1β Levels in HGHL HepG2 Cells

Levels of cAMP and IL-1β were determined using ELISA. The levels of cAMP in the model group were elevated compared to those in the normal group (*P* < 0.001). In contrast, cAMP levels in the JTD (3 mg/mL, 15 mg/mL) and Met groups were lower than in the model group (*P* < 0.001). No difference in cAMP levels was observed between the JTD (15 mg/mL) and Metformin groups; however, both groups exhibited significantly lower cAMP levels than the JTD (3 mg/mL) group (Fig. **9F**). These findings suggest that JTD inhibits gluconeogenesis in HepG2 cells by suppressing cAMP levels.

The levels of IL-1β in the model group were elevated compared to those in the control group (*P* < 0.001). In comparison with the model group, both JTD groups (3 mg/mL and 15 mg/mL) reduced IL-1β levels (*P* < 0.001), with no significant difference observed between the two JTD groups. The IL-1β level in the Met group was comparable to that in the model group (*P* > 0.05, *P* = 2.355) (Fig. **9G**). These findings suggest that JTD suppresses the inflammatory response in HepG2 cells exposed to high glucose and lipids by reducing IL-1β levels.

### 3.12 JTD Increased PRKAB1 Expression in HGHL HepG2 Cells

**Fig. (10):**
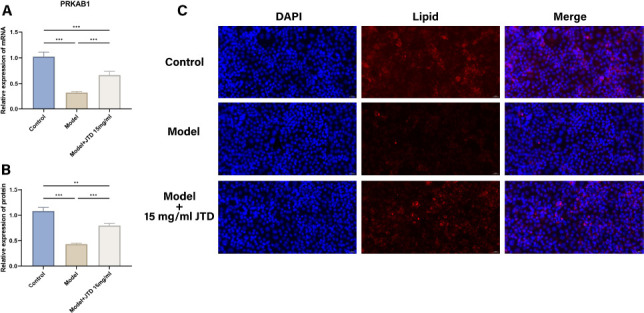
Effect of JTD on PRKAB1 *in vitro*. JTD intervention increased the mRNA expression level of PRKAB1 (**A**). JTD intervention also enhanced the protein expression level of PRKAB1 (**B** and **C**). *p < 0.05, **p < 0.01, ***p < 0.001.

The results indicated that PRKAB1 mRNA expression was reduced in the model group compared to the control group, whereas JTD treatment (15 mg/mL) markedly increased PRKAB1 mRNA levels (*P* < 0.001) (Fig. **10A**). A similar trend was observed for PRKAB1 protein expression, as evidenced by immunofluorescence results (Figs. **10B** and **C**).

## 4 DISCUSSION

T2DM and NAFLD pose significant health challenges with overlapping pathophysiology [35-37]. JTD, a traditional Chinese medicine formula composed of *Atractylodes macrocephala*, *Scrophularia ningpoensis*, *Astragalus membranaceus*, *Rehmannia glutinosa*, *Pueraria lobata*, and *Salvia miltiorrhiza,* has demonstrated potential hypoglycemic and hypolipidemic effects after years of clinical application. However, its underlying molecular mechanisms, particularly the synergistic interactions between T2DM and NAFLD, remain poorly understood.

This study leverages a novel integrated approach, combining network pharmacology with SMR and molecular docking, to move beyond association and identify causal molecular targets of JTD implicated in both conditions. The integration of eQTL and pQTL analyses identified eight shared genetically predicted causal genes (*ADAMTS4, ALDH2, GLO1, CDH1, PDHB, PRKAB1, TCF4,* and *EP300*) associated with both T2DM and NAFLD. These genes are implicated in critical processes such as inflammatory responses, oxidative stress, and endoplasmic reticulum stress, all of which are closely linked to impaired insulin secretion and the progression of IR. The eight shared genes identified in this study indicate that JTD targets common disease mechanisms in T2DM and NAFLD, including insulin resistance, oxidative stress, and lipid metabolism dysfunction. *PRKAB1* is a key target: molecular docking shows strong binding between *PRKAB1* and Salsalate, a component of JTD, and *in vitro* tests confirm that JTD restores *PRKAB1* expression in HepG2 cells under HGHL conditions, suggesting it helps regulate AMPK-mediated energy balance. *ALDH2*, *GLO1*, and *PDHB* form a gene cluster crucial for maintaining redox balance and regulating glucose-lipid interactions. Their roles—*ALDH2* in detoxifying aldehydes and protecting mitochondria, *GLO1* in reducing glycotoxicity, and PDHB in pyruvate oxidation—highlight JTD’s ability to reduce oxidative stress and metabolic dysfunction in both diseases. *TCF4* and *EP300* connect gene regulation with metabolic effects: *TCF4* affects β-cell function and glucose metabolism through Wnt signaling, while EP300 influences liver fat buildup *via* epigenetic changes. *CDH1* and *ADAMTS4* support tissue structure—*CDH1* maintains epithelial integrity, and *ADAMTS4* controls ECM turnover to reduce fibrosis. Table **1** summarizes the roles of these genes, supporting JTD’s multi-target approach in treating the comorbidity of T2DM and NAFLD. These findings suggest that JTD's therapeutic effects on T2DM-NAFLD may be mediated *via* the regulation of these shared targets and related mechanisms. The eight shared genes were identified through independent screening and intersection verification, demonstrating their unbiased causal links to both T2DM and NAFLD. This supports the hypothesis that JTD targets common mechanisms to treat both diseases. Furthermore, molecular docking predicted a strong interaction between PRKAB1 and Salsalate, a component of JTD. Consistent with these predictions, *in vitro* experiments using the HGHL-induced HepG2 cell model confirmed that JTD suppresses hepatic gluconeogenesis, reduces lipid accumulation, and downregulates IL-1β expression, indicating its potential to modulate liver metabolism. However, further studies in more physiologically relevant models are needed to validate these findings. Additionally, JTD was shown to reverse the suppression of PRKAB1 expression caused by HGHL in hepatocytes. These findings provide robust evidence for JTD-regulated shared molecular pathways and establish a mechanistic basis for its potential efficacy in managing the comorbidity of T2DM and NAFLD. Among these shared targets, *PRKAB1*, encoding the β-1 non-catalytic subunit of AMPK, is particularly noteworthy.

Both T2DM and NAFLD are characterized by insulin resistance and impaired metabolism [38-40]; yet, direct evidence linking PRKAB1 to T2DM remains limited. In NAFLD, however, studies have shown that PRKAB1 downregulation contributes to impaired lipid metabolism and hepatic steatosis, while its upregulation (*e.g.*, *via* miR-802 inhibition) activates the AMPK pathway to improve NAFLD symptoms [34]. Despite the lack of direct evidence of T2DM, PRKAB1’s role in AMPK activation underscores its potential in regulating metabolic pathways relevant to both conditions [41, 42]. Our SMR analysis found a genetic link between PRKAB1 and the risks of T2DM and NAFLD. Molecular docking showed strong binding (-7.043 kcal/mol) between PRKAB1 and Salsalate, a component of JTD. *In vitro* tests confirmed that JTD reverses PRKAB1 downregulation caused by HGHL, suggesting its potential as a key target. However, as the β1 subunit of AMPK, it is unclear whether PRKAB1 activates the AMPK pathway. Since AMPKα phosphorylation, a marker of AMPK activity, was not measured, the results cannot confirm if JTD works through PRKAB1. Further studies are needed to clarify its role in the effects of JTD. Metabolic genes are essential in the common pathogenesis underlying both T2DM and NAFLD [43]. The causal involvement of the metabolic gene cluster (*ALDH2, PDHB,* and *GLO1*) further reinforces the link between JTD's targets and core metabolic disturbances shared between T2DM and NAFLD. ALDH2 detoxifies acetaldehyde and other aldehydes, indirectly supporting energy metabolism through the maintenance of mitochondrial function and the reduction of oxidative stress [44]. PDHB, a key component of the pyruvate dehydrogenase complex, catalyzes the conversion of pyruvate to acetyl-CoA, a critical step in glucose metabolism and energy production [45]. GLO1 detoxifies methylglyoxal, a byproduct of glycolysis, and also links it to energy metabolism and oxidative stress [46, 47]. Recent studies have emphasized the role of these genes in the onset and advancement of T2DM and NAFLD. For example, ALDH2 mutations reduce enzymatic activity [48, 49], increasing the risk of T2DM through impaired glucose homeostasis and insulin resistance, while also potentially raising the risk of NAFLD by altering toxic aldehyde metabolism in carriers of the mutant allele [50, 51]. On the other hand, *PDHB* was upregulated in NAFLD and associated with clinical features such as steatosis [52], while its genetic variants were also linked to T2DM through their impact on glucose metabolism [53]. The *GLO1* gene is implicated in both T2DM and NAFLD. Elevated methylglyoxal levels, a substrate of GLO1, contribute to insulin resistance and NAFLD progression by inducing oxidative stress and protein glycation, while GLO1 downregulation exacerbates these effects [54]. Upregulating GLO1 activity, as demonstrated in clinical trials, can enhance insulin sensitivity and mitigate inflammation, underscoring its potential as a therapeutic target for both diseases [54]. In this research, it was discovered that the levels of these three proteins were causally linked to both conditions, suggesting that they may be involved in the shared pathogenic mechanisms underlying T2DM and NAFLD. This highlights the interconnected nature of metabolic pathways in the pathogenesis of T2DM and NAFLD, underscoring the potential for therapeutic strategies targeting these metabolic genes to address the common underlying mechanisms of these comorbid conditions.

*TCF4* serves as a transcription factor with a key role in the Wnt signaling pathway, whereas p300 is a histone acetyltransferase that modulates gene transcription epigenetically through chromatin structure modification [55, 56]. Variations in the *TCF4* gene are strongly associated with an increased risk of T2DM, primarily by regulating genes involved in glucose metabolism and insulin secretion, thereby impairing pancreatic β-cell function [57]. While its role in NAFLD is less direct, the influence of *TCF4* on glucose metabolism may indirectly impact liver health due to the metabolic interconnections between Type 2 Diabetes Mellitus (T2DM) and NAFLD. *EP300*, another shared target, is a key regulator in T2DM, contributing to β-cell dysfunction and insulin resistance *via* mechanisms such as promoting VDAC1 overexpression and impairing mitochondrial function in β-cells [58]. In the context of NAFLD, EP300 activation has been shown to promote lipid accumulation in hepatocytes by modulating mTORC1 activity and autophagy [59-61]. The SMR analyses corroborated the causal involvement of both *TCF4* and *EP300* in the risk of T2DM and NAFLD.

The *ADAMTS4* gene encodes a metalloproteinase that degrades proteoglycans, which are crucial components of the extracellular matrix (ECM). Despite the limited direct research on the connection between *ADAMTS4* and type 2 diabetes mellitus (T2DM), evidence from studies suggests that ADAMTS4 may be involved in diabetes-related atherosclerosis. Increased serum concentrations of ADAMTS4 have been detected in patients with diabetes, particularly those with atheroma plaques, indicating its potential involvement in vascular complications associated with diabetes [62]. On the other hand, *ADAMTS4* expression was downregulated in NAFLD, potentially contributing to the accumulation of ECM components and fibrosis in the liver [63]. This study found that ADAMTS4 protein levels in blood are positively associated with NAFLD risk, differing from the liver mRNA patterns observed in previous studies. This difference may result from tissue heterogeneity and methodological variations. ADAMTS4 in the liver, as a matrix metalloproteinase, may help degrade the extracellular matrix, and its down-regulation could promote fibrosis. In contrast, circulating ADAMTS4 may reflect systemic metabolic inflammation, with higher levels indicating a pro-inflammatory state. These roles suggest distinct mechanisms of NAFLD [63]. This study utilized blood pQTL data, whereas most others employed liver mRNA data. GTEx liver eQTL data indicate that ADAMTS4 mRNA expression in the liver is negatively correlated with NAFLD, which contrasts with the blood-based result obtained in this study. This supports the tissue-specific regulation of ADAMTS4 [26]. Overall, the role of ADAMTS4 in NAFLD should be studied separately in the liver and blood.

The functional enrichment analysis highlighted that the shared GO terms between T2DM and NAFLD-associated genes, including membrane microdomain, oxidoreductase activity, response to nutrient levels, and response to decreased oxygen levels, may reflect common pathological mechanisms. Membrane microdomains, which are crucial for lipid metabolism and insulin signaling, can lead to lipid accumulation in NAFLD and IR in T2DM when dysfunctional [64-66]. Oxidoreductase activity, involved in oxidative stress and mitochondrial function, can cause liver cell damage in NAFLD and exacerbate insulin resistance in T2DM [67-69]. Abnormal responses to nutrient levels can disrupt lipid homeostasis in NAFLD and impair insulin sensitivity in type 2 diabetes mellitus (T2DM) [70, 71]. Lastly, the response to decreased oxygen levels, often seen in NAFLD progression and T2DM, can drive inflammation and metabolic dysfunction *via* the HIF-1 pathway [72-74]. These shared pathways highlight the interconnected nature of NAFLD and T2DM, suggesting potential therapeutic targets for further research into the molecular mechanisms of these diseases.

This study presents a systematic exploration of T2DM and NAFLD-related genes targeted by JTD, employing network pharmacology, SMR analysis, and molecular docking methods [75, 76]. The integrated use of SMR and network pharmacology has improved the accuracy of target identification, revealed common mechanisms of action, and offered insights into the interaction strength and binding modes between JTD components and target proteins through molecular docking. A simple unidirectional causality does not link T2DM and NAFLD; instead, they interact in a complex, bidirectional manner. T2DM promotes hepatic lipid accumulation through hyperglycemia and insulin resistance, while NAFLD exacerbates insulin resistance *via* the liver-pancreas axis and inflammatory cytokine release. The eight shared genes identified in this study (*e.g.*, PRKAB1, ALDH2), along with enriched pathways such as redox homeostasis and nutrient response, likely reflect the shared pathological basis of both diseases rather than a one-way regulatory mechanism. These findings suggest that JTD, as a multi-component formulation, exerts synergistic effects on T2DM and NAFLD by targeting common pathogenic mechanisms. These shared targets may serve as potential biomarkers or novel therapeutic intervention points for the comorbidity of T2DM and NAFLD.

However, several limitations merit attention. First, the exclusive reliance on *Cis*-QTLs in SMR analysis may limit the scope of identified genetic associations, potentially overlooking long-range regulatory mechanisms that play critical roles in T2DM and NAFLD pathogenesis, such as distal enhancers or trans-acting variants involved in modulating hepatic metabolic pathways [77-79]. Second, the generalizability of the findings is limited by the use of datasets derived exclusively from individuals of European ancestry; differences in genetic architecture and disease mechanisms across populations may restrict the applicability of these results to non-European groups [80]. Third, the “causal signals” identified by SMR in this study are based on the inference of genetic association. Subsequent molecular docking and *in vitro* experiments provided preliminary support for these associations, but the true biological causal relationship still needs to be confirmed through *in vivo* studies. Although *in vitro* tests with HGHL-induced HepG2 cells provide early insights, showing that PRKAB1 binds most strongly to JTD and that JTD restores its expression, suggesting a key therapeutic role, the unclear mechanism of PRKAB1 in the AMPK pathway remains a major limitation. Further studies are needed to clarify its role and the regulatory mechanisms involved. The lack of *in vivo* models, such as diet-induced rodent models of T2DM-NAFLD comorbidity, limits the translational value of the study’s findings. While the HepG2 cell line used in this study can mimic liver metabolic disorders caused by high glucose levels, it has key limitations. As a cancer-derived cell line, HepG2 cells exhibit a metabolic profile (*e.g.*, insulin sensitivity and lipid synthesis) that differs from that of normal hepatocytes and lack essential functions such as glycogen storage and bile secretion. Moreover, *in vitro* models cannot reflect systemic or tissue-specific metabolic responses and inter-organ interactions—central features of T2DM and NAFLD development. In addition, this study focused on short-term effects (24 hours) and did not assess long-term cellular adaptations. Therefore, the hypoglycemic, lipid-lowering, and PRKAB1 regulatory effects of JTD observed *in vitro* should be validated in primary human hepatocytes and T2DM-NAFLD animal models. Combining *in vivo* and *in vitro* approaches would improve the reliability and broader applicability of the results.

## CONCLUSIONS

This research, which integrates network pharmacology, SMR, and molecular docking with *in vitro* validation, identifies eight shared causal target genes (ADAMTS4, ALDH2, GLO1, CDH1, PDHB, PRKAB1, TCF4, and *EP300*) for JTD in T2DM and NAFLD. JTD may exert synergistic therapeutic effects by targeting shared mechanisms, such as insulin resistance, dysregulation of lipid metabolism, and oxidative stress. This study highlights the value of targeting shared pathological mechanisms in comorbid metabolic diseases. It provides a rigorous basis for future research on personalized JTD-based treatments, with a focus on modulators like PRKAB1.

## Figures and Tables

**Table 1:** Summary table of eight shared genetically predicted causal genes.

**Gene**	**Primary Function**	**Association with T2DM**	**Association with NAFLD**	**Relevance to JTD Components / Pathways**
PRKAB1	Encodes the β1 subunit of AMPK, regulating energy metabolism and lipid homeostasis	Genetically linked to T2DM risk *via* SMR; JTD restores its expression in HGHL-induced cells	Downregulation contributes to hepatic steatosis; JTD upregulates it to improve lipid metabolism	Strong binding affinity with Salsalate (JTD component); modulates AMPK-mediated gluconeogenesis and lipid oxidation
ALDH2	Detoxifies aldehydes, maintains mitochondrial function, and reduces oxidative stress	Mutations impair glucose homeostasis and insulin resistance, increasing T2DM risk	Dysregulation exacerbates hepatic oxidative stress and steatosis	Targeted by JTD to mitigate oxidative stress, a shared pathogenic mechanism in both diseases
GLO1	Detoxifies methylglyoxal (a glycolysis byproduct), reducing glycation and oxidative stress	Downregulation linked to insulin resistance and hyperglycemia in T2DM	Elevated methylglyoxal promotes hepatic inflammation and fibrosis	JTD may upregulate GLO1 to alleviate glycotoxicity and oxidative damage in both conditions
CDH1	Encodes E-cadherin, regulating cell adhesion and epithelial integrity	Implicated in pancreatic β-cell dysfunction and insulin secretion impairment	Dysregulation associated with hepatic fibrosis and inflammation	Involved in JTD’s modulation of tissue integrity pathways shared between liver and pancreatic tissues
PDHB	Key subunit of pyruvate dehydrogenase, critical for glucose oxidation and energy production	Variants affect glucose metabolism, increasing T2DM risk	Upregulated in NAFLD, linked to hepatic steatosis and mitochondrial dysfunction	JTD may target PDHB to improve glucose-lipid metabolic crosstalk in the liver
TCF4	Transcription factor in Wnt signaling, regulating glucose metabolism genes	Variants associated with β-cell dysfunction and impaired insulin secretion	Indirectly influences hepatic metabolism *via* systemic glucose regulation	JTD modulates Wnt signaling to improve glucose homeostasis in both T2DM and NAFLD
EP300	Histone acetyltransferase, regulating gene transcription in metabolism and inflammation	Promotes β-cell dysfunction *via* mitochondrial impairment in T2DM	Activates mTORC1 to enhance hepatic lipid accumulation and fibrosis	Targeted by JTD to suppress inflammatory and lipogenic pathways in the liver
ADAMTS4	Metalloproteinase degrading extracellular matrix (ECM) components	Indirectly linked to vascular complications in diabetes	Downregulation promotes hepatic ECM accumulation and fibrosis	JTD may regulate ADAMTS4 to reduce systemic inflammation and hepatic fibrosis

## Data Availability

The GWAS summary statistics for T2DM and NAFLD can be sourced from the FinnGen database.

## LIST OF ABBREVIATIONS

JTD: Jiangtang Decoction
NAFLD: Non-Alcoholic Fatty Liver Disease
SMR: Summary-data Mendelian Randomization
HGHL: High-Glucose and High-Lipid
T2DM: Type 2 Diabetes Mellitus
TCM: Traditional Chinese Medicine
ECM: Extracellular Matrix
