# Supplementary material for: Jiangtang Decoction for Type 2 Diabetes and NAFLD: Integrative Analysis via Network Pharmacology, Mendelian Randomization, Molecular Docking, and In Vitro Validation
Source: Endocr Metab Immune Disord Drug Targets. 2025 Aug 22;26:E18715303424915. doi: 10.2174/0118715303424915250818045151 (PMC13334248; doi:10.2174/0118715303424915250818045151)
Supplement: Supplementary file 1 [file EMIDDT-26-E18715303424915_SD1.pdf]

Supplementary Material

Jiangtang Decoction for Type 2 Diabetes and NAFLD: Integrative Analysis via Network Pharmacology, Mendelian Randomization, Molecular Docking, and In Vitro Validation

Wenbo Gong[1], Xueke Lu[1,2] and Siying Weng[1,2,*]

[1]Ningbo Municipal Hospital of TCM, Affiliated Hospital of Zhejiang Chinese Medical University, Ningbo, China; [2]The Affiliated Joint Training Base of Zhejiang Chinese Medical University, Ningbo, China

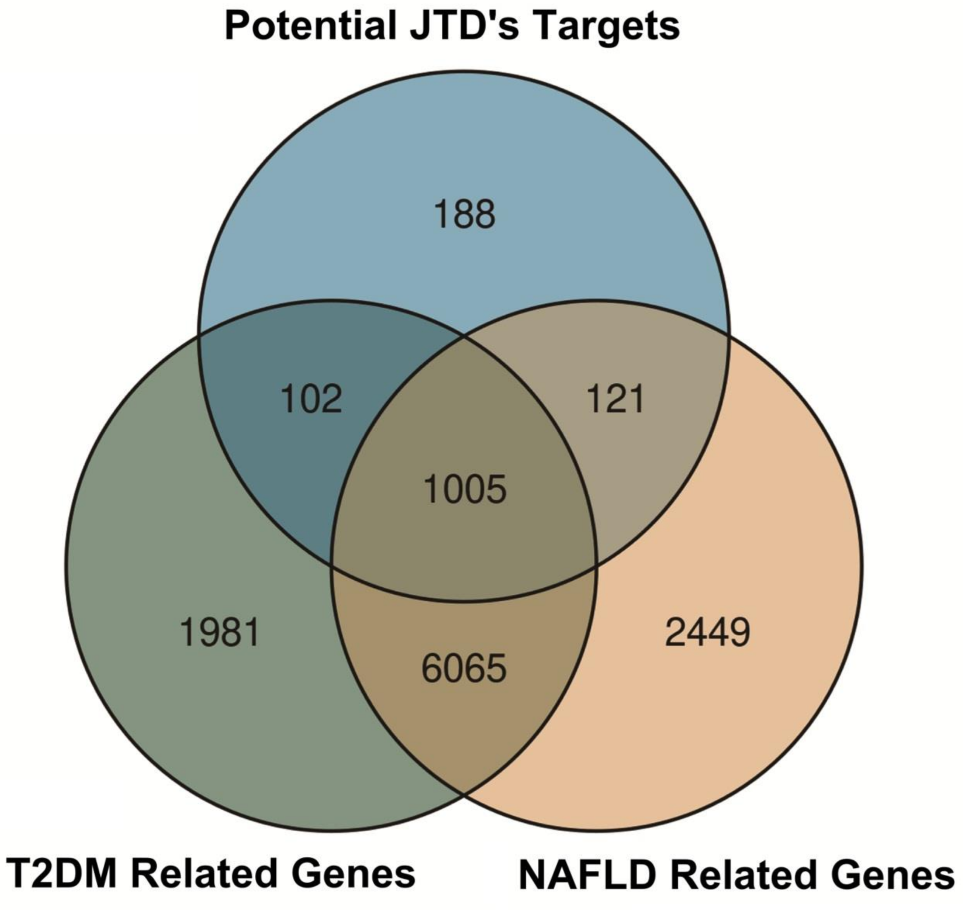

Fig. (S1). A Venn diagram showing the overlapping genes between JTD target genes and T2DM or NAFLD-related genes.

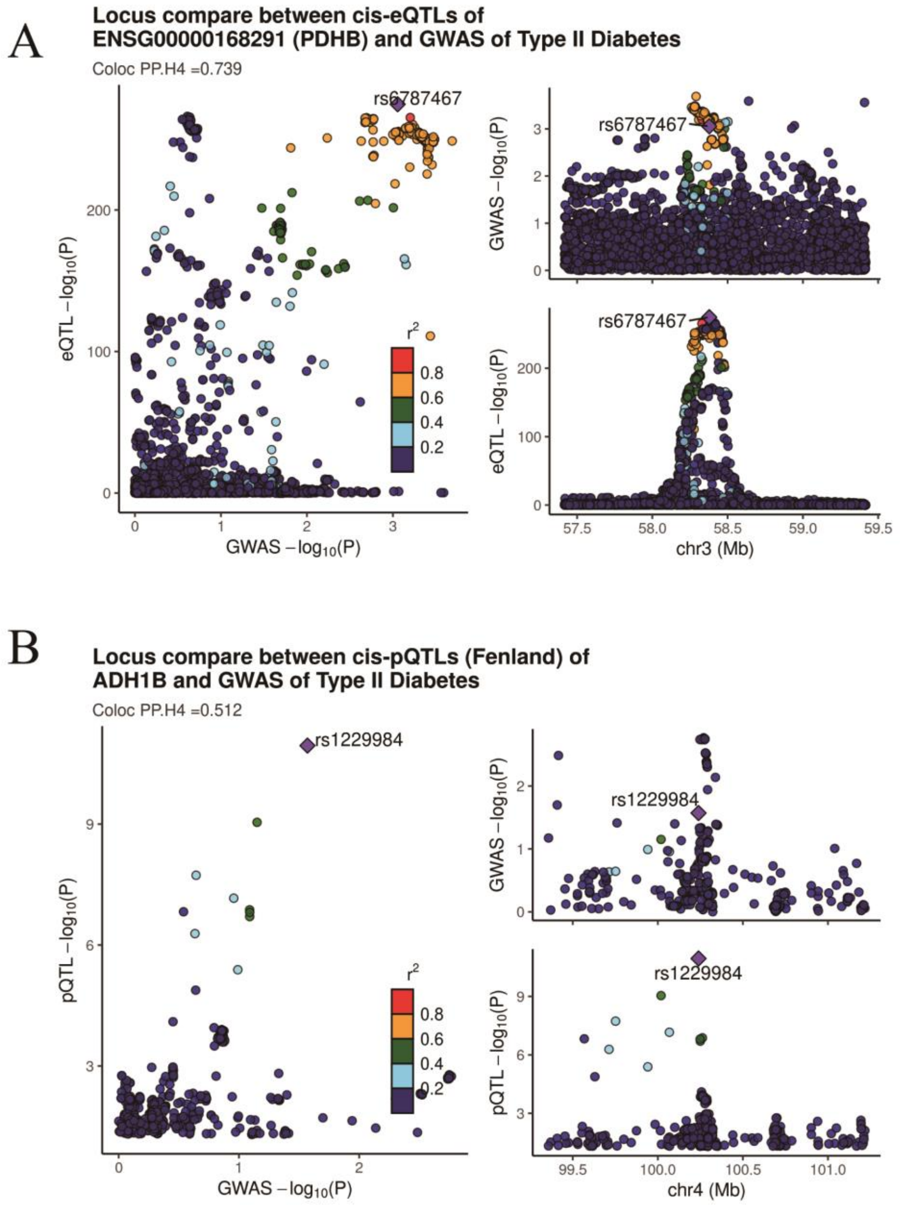

Fig. (S2). Rrepresentative plots of colocalization analysis.

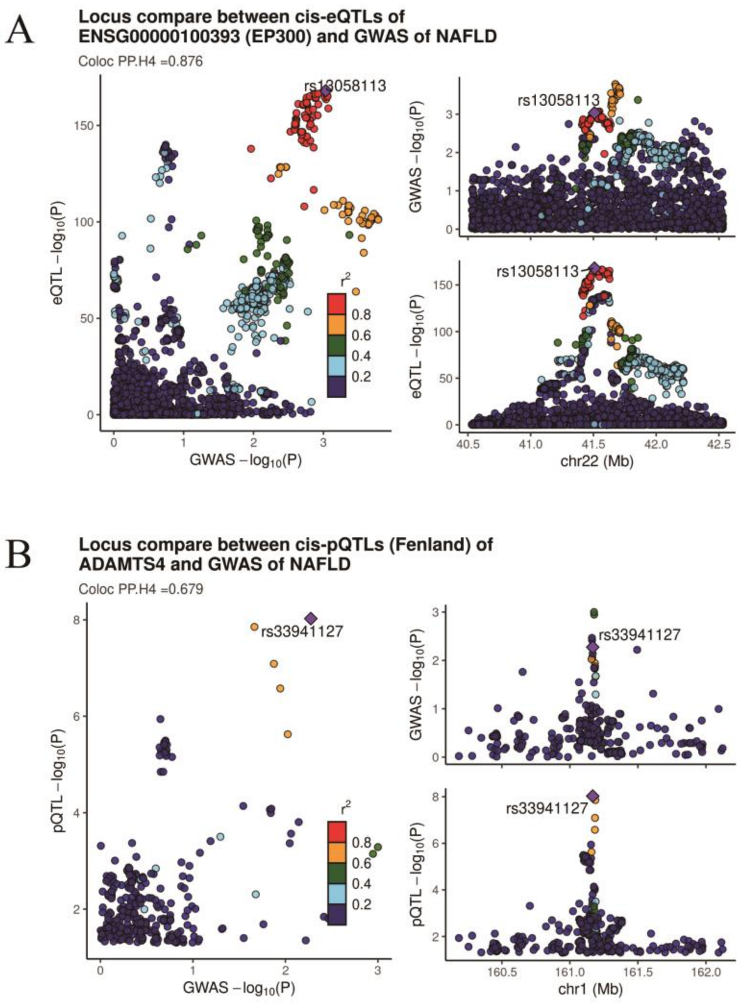

Fig. (S3). Representative plots of colocalization analysis.
